# Structural insights into the distinct ligand recognition and signaling of the chemerin receptors CMKLR1 and GPR1

**DOI:** 10.1093/procel/pwae073

**Published:** 2025-01-03

**Authors:** Xiaowen Lin, Lechen Zhao, Heng Cai, Xiaohua Chang, Yuxuan Tang, Tianyu Luo, Mengdan Wu, Cuiying Yi, Limin Ma, Xiaojing Chu, Shuo Han, Qiang Zhao, Beili Wu, Maozhou He, Ya Zhu

**Affiliations:** School of Pharmaceutical Science and Technology, Hangzhou Institute for Advanced Study, University of Chinese Academy of Sciences, Hangzhou 310024, China; State Key Laboratory of Drug Research, State Key Laboratory of Chemical Biology, Shanghai Institute of Materia Medica, Chinese Academy of Sciences, Shanghai 201203, China; University of Chinese Academy of Sciences, Beijing 100049, China; School of Pharmaceutical Science and Technology, Hangzhou Institute for Advanced Study, University of Chinese Academy of Sciences, Hangzhou 310024, China; University of Chinese Academy of Sciences, Beijing 100049, China; School of Pharmaceutical Science and Technology, Hangzhou Institute for Advanced Study, University of Chinese Academy of Sciences, Hangzhou 310024, China; Lingang Laboratory, Shanghai 200000, China; School of Life Science and Technology, ShanghaiTech University, Shanghai 201210, China; School of Pharmaceutical Science and Technology, Hangzhou Institute for Advanced Study, University of Chinese Academy of Sciences, Hangzhou 310024, China; University of Chinese Academy of Sciences, Beijing 100049, China; School of Pharmaceutical Science and Technology, Hangzhou Institute for Advanced Study, University of Chinese Academy of Sciences, Hangzhou 310024, China; University of Chinese Academy of Sciences, Beijing 100049, China; School of Pharmaceutical Science and Technology, Hangzhou Institute for Advanced Study, University of Chinese Academy of Sciences, Hangzhou 310024, China; University of Chinese Academy of Sciences, Beijing 100049, China; School of Pharmaceutical Science and Technology, Hangzhou Institute for Advanced Study, University of Chinese Academy of Sciences, Hangzhou 310024, China; University of Chinese Academy of Sciences, Beijing 100049, China; State Key Laboratory of Drug Research, State Key Laboratory of Chemical Biology, Shanghai Institute of Materia Medica, Chinese Academy of Sciences, Shanghai 201203, China; State Key Laboratory of Drug Research, State Key Laboratory of Chemical Biology, Shanghai Institute of Materia Medica, Chinese Academy of Sciences, Shanghai 201203, China; State Key Laboratory of Drug Research, State Key Laboratory of Chemical Biology, Shanghai Institute of Materia Medica, Chinese Academy of Sciences, Shanghai 201203, China; State Key Laboratory of Drug Research, State Key Laboratory of Chemical Biology, Shanghai Institute of Materia Medica, Chinese Academy of Sciences, Shanghai 201203, China; University of Chinese Academy of Sciences, Beijing 100049, China; State Key Laboratory of Drug Research, State Key Laboratory of Chemical Biology, Shanghai Institute of Materia Medica, Chinese Academy of Sciences, Shanghai 201203, China; University of Chinese Academy of Sciences, Beijing 100049, China; Zhongshan Institute for Drug Discovery, Shanghai Institute of Materia Medica, Chinese Academy of Sciences, Zhongshan 528400, China; School of Pharmaceutical Science and Technology, Hangzhou Institute for Advanced Study, University of Chinese Academy of Sciences, Hangzhou 310024, China; State Key Laboratory of Drug Research, State Key Laboratory of Chemical Biology, Shanghai Institute of Materia Medica, Chinese Academy of Sciences, Shanghai 201203, China; University of Chinese Academy of Sciences, Beijing 100049, China; School of Life Science and Technology, ShanghaiTech University, Shanghai 201210, China; School of Pharmaceutical Science and Technology, Hangzhou Institute for Advanced Study, University of Chinese Academy of Sciences, Hangzhou 310024, China; University of Chinese Academy of Sciences, Beijing 100049, China; Lingang Laboratory, Shanghai 200000, China

**Dear Editor,**

Chemerin functions as both an adipocytokine and a chemotactic factor and plays key roles in adipogenesis and inflammation (Bozaoglu et al., 2007; Bondue et al., 2011). The chemerin receptors chemokine-like receptor 1 (CMKLR1) and G protein-coupled receptor 1 (GPR1), play crucial roles in regulating obesity, inflammation, and cancer, highlighting their potential as therapeutic targets (Bozaoglu et al., 2007; Ernst and Sinal, 2010; Su et al., 2021). Although both receptors recognize the same ligand, chemerin, and share high sequence identity, they exhibit distinct signaling properties and biological functions (Bozaoglu et al., 2007; De Henau et al., 2016; Fischer et al., 2021). CMKLR1 is the canonical chemerin receptor that mediates both G protein and arrestin signaling, while GPR1 exhibits weak G protein signaling but strong arrestin-dependent and -independent internalization (De Henau et al., 2016; Fig. 1A and 1B). The chemerin-CMKLR1 axis regulates lipid and glucose metabolism (Kennedy and Davenport, 2018), and is associated with chronic inflammation, obesity, and obesity-related disorders such as insulin resistance and metabolic syndrome, with several compounds and antibodies under preclinical or early clinical investigation (Goralski et al., 2007; Kennedy et al., 2016; Trilleaud et al., 2021). In contrast, the mechanisms of atypical signaling and biological function of GPR1 remain unclear.

**Figure 1.**
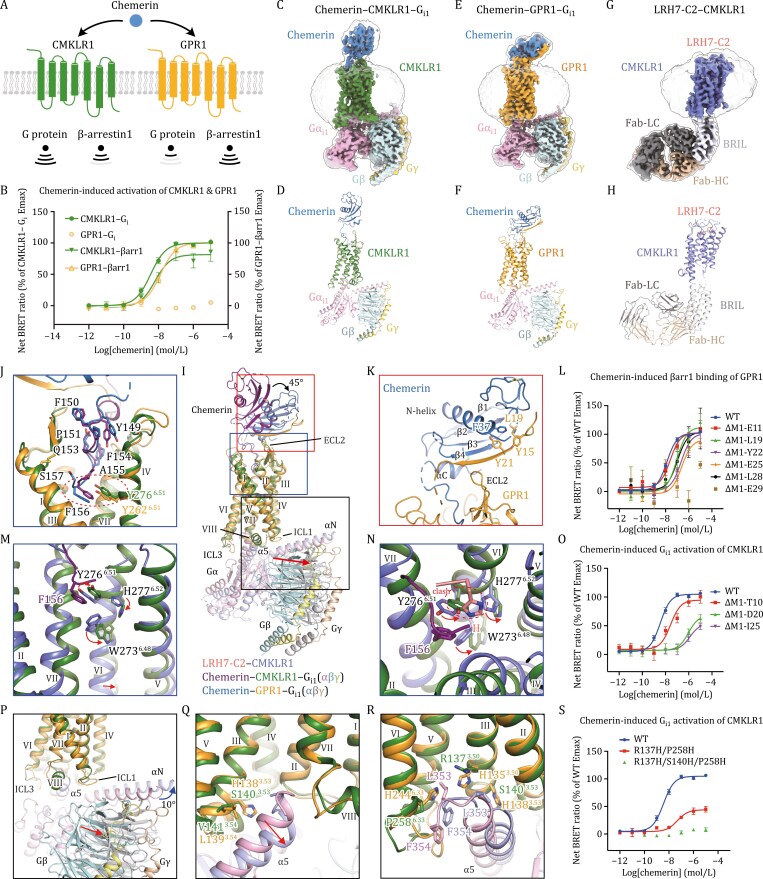
**Distinct molecular mechanism underlying the ligand recognition and receptor activation of the chemerin receptors CMKLR1 and GPR1.** (A and B) Chemerin induced distinct G protein and β-arrestin1 signaling of CMKLR1 and GPR1. (C–H) The cryo-EM maps and structures of the CMKLR1 and GPR1 complexes. Unsharpened maps are shown in transparency to indicate the transmembrane region surrounded by the detergent micelle densities. The maps and structures are colored according to chains. The chemerin, active CMKLR1, inactive CMKLR1, GPR1, Gα_i1_, Gβ, Gγ, LRH7-C2, BRIL, the light chain (Fab-LC) and heavy chain (Fab-HC) of anti-BRIL Fab are labled directly in the figure and colored sky blue, forest, slate, bright orange, pink, pale cyan, yellow, salmon, white, gray and wheat, respectively. The structures are shown in cartoon representation, except that LRH7-C2 is shown as sticks. (C and D) The cryo-EM maps and structures of the chemerin–CMKLR1–G_i1_ complex. (E and F) The cryo-EM maps and structures of the chemerin–GPR1–G_i1_ complex. (G and H) The cryo-EM maps and structures of the inactive CMKLR1 bound to the antagonist LRH7-C2. (I) Overall comparison of the chemerin–CMKLR1–G_i1_ and chemerin–GPR1–G_i1_ structures. The main interaction sites are indicated by red, blue, and black boxes, respectively. The black arrow indicates the movement of the chemerin core in the GPR1 structure relative to the CMKLR1 structure. The red arrow indicates the movement of the Gβ in the GPR1 structure relative to the CMKLR1 structure. (J) The binding site of the chemerin C-terminus in CMKLR1 and GPR1. The side chain of chemerin C-terminal residues Y149–S157 are shown as sticks. The hydrophobic interactions between F156 of chemerin and Y^6.51^ of CMKLR1/GPR1 are indicated by the red dashed oval. (K) Interactions between the chemerin core domain and the GPR1 N-terminus. The chemerin and receptor residues involved in interactions are shown as sticks. (L) Chemerin-induced βarr1 binding of the GPR1 N-terminal truncation measured by bioluminescence resonance energy transfer (BRET) assay. (M and N) Side view (M) and top view (N) of structural superposition of CMKLR1 in active and inactive states. The residues involved in signal transduction upon chemerin binding are shown as sticks. The red arrows indicate the conformational changes of residues from inactive conformation to active conformation. The red dashed oval shows the histidine residue at the N-terminus of LRH7-C2 forms a steric clash with Y256^6.51^ of CMKLR1 in chemerin–CMKLR1–G_i1_ structure. (O) Chemerin-induced G_i1_ activation of CMKLR1 measured by BRET assays. (P) Comparison of G protein between CMKLR1 and GPR1 complex structures. The straight red arrow indicates the movement of the Gβ subunit in the GPR1 structure relative to the CMKLR1 structure. The bent blue arrow indicates the rotation of the αN helix in the GPR1 structure relative to the CMKLR1 structure. (Q and R) Binding modes of the Gα_i1_ α5 helix in CMKLR1 and GPR1. (Q) The receptor residues at positions 3.53 and 3.54 that may account for the different binding modes of the α5 helix are shown as sticks. The red arrow indicates the shift of the α5 helix in the GPR1 structure relative to the CMKLR1 structure. (R) The receptor residues that may determine the receptor ability of activating the G proteins are shown as sticks. (S) The CMKLR1 mutants of the residues that may determine the receptor ability of activating the G proteins by BRET assays. All functional data are shown as mean ± SEM from at least three independent experiments performed in technical duplicate. Table S1 provides detailed independent experiment numbers (*n*), statistical evaluation and expression levels.

Chemerin activation requires proteolytic cleavage of its inactive precursor, pre-prochemerin (163 amino acids), to release the active form (residues 21–157; Buechler et al., 2019). It has been reported that the C-terminal fragment of chemerin is crucial for receptor activation (Zhao et al., 2022). A synthetic peptide known as chemerin 9 (C9) that corresponds to the residues 149–157 in the C-terminus of chemerin can activate CMKLR1, albeit with 40-fold lower activity compared to that of the full-length active form of chemerin (residues 21–157; Shimamura et al., 2009). This discrepancy suggests a vital role of the chemerin core region in receptor recognition and activation. Although the structures of the C9-bound CMKLR1 and GPR1, as well as the full-length chemerin-bound GPR1, have been reported (Liu et al., 2024; Wang et al., 2023; Zhang et al., 2023), the molecular mechanisms underlying the full-length chemerin recognition of CMKLR1 and the diverse signaling properties of the two chemerin receptors remain to be fully elucidated.

Thus, we solved the structures of these two receptors bound to the full-length chemerin and G_i1_ protein. Aiming to improve protein stability and yield, the residues L323–Q355 at the C-terminus of GPR1 were truncated. The wild-type CMKLR1 or the C-terminally truncated GPR1 was co-expressed with chemerin and G_i1_ protein, then the complexes were purified and analyzed using cryo-electron microscopy (cryo-EM) single-particle analysis, yielding density maps at overall resolutions of 3.5 Å and 3.6 Å for the chemerin–CMKLR1–G_i1_ and chemerin–GPR1–G_i1_ complexes, respectively (Figs. 1C–F, S1A–F, S2A and S2B; Table S2). To better understand the receptor activation mechanism, the structure of CMKLR1 in complex with the antagonistic peptide LRH7-C2 (His-(D)Trp-Asn-Thr-Val-Val-Ser-NH_2_; Jian et al., 2017), was also solved. To allow structure determination, the residues R251-K254 in the third intracellular loop (ICL3) of CMKLR1 were replaced with the apocytochrome b562 (BRIL) fusion protein. The rigid connection linker from A_2A_AR and an anti-BRIL Fab were used to facilitate the particle alignment in cryo-EM data processing (Tsutsumi et al., 2020). Furthermore, the mutation F259^6.43^D, which potentially forms a salt bridge with the R137^3.50^ to limit the conformational change of helix VI, was introduced, and the C-terminal residues S339–L373 of CMKLR1 were truncated to improve protein quality. The cryo-EM analysis yielded a map of the LRH7-C2–CMKLR1 complex at 3.9 Å resolution (Figs. 1G, 1H, S1G–I and S2C; Table S2). The cryo-EM maps allowed unambiguous placement of most of the residues in chemerin, CMKLR1, GPR1, G_i1_ protein, BRIL, and the anti-BRIL Fab (Fig. S3A–C). The densities of the antagonist LRH7-C2 were only observed for its N-terminus (Fig. S3C).

Consistent with the differential signaling properties of CMKLR1 and GPR1, our structures reveal distinct binding patterns of chemerin to these two receptors (Fig. 1I). Similar to the previously reported structure of the C9-bound CMKLR1 (Wang et al., 2023), the chemerin-bound CMKLR1 and GPR1 exhibit a ligand binding pocket within the receptor helical bundle for the C-terminus of chemerin (Fig. 1I and 1J). However, the relative orientation between the core region of chemerin and the receptor helical bundle differs in the structures of CMKLR1 and GPR1 (Fig. 1I). In the chemerin–CMKLR1–G_i1_ structure, the long axis of the chemerin core is perpendicular to the membrane plane, while in the chemerin–GPR1–G_i1_ structure, the ligand core shifts towards the second extracellular loop (ECL2) with its long axis forming a 45° angle with the membrane plane (Fig. 1I).

Supported by a previous study (Zhao et al., 2022) and our functional data, the chemerin exhibits much higher agonistic activity in inducing CMKLR1 and GPR1 activation than the C9 peptide (Table S1), suggesting extra contacts between the chemerin core and receptor in addition to the interactions mediated by the agonist C-terminus. Indeed, in the structures of chemerin–GPR1–G_i1_, the receptor N-terminus makes extensive interactions with the core region of chemerin, with its residues D20–D27 running anti-parallelly with the residues L111–C117 in chemerin, forming a compact β-sheet (Fig. 1K). In addition, the side chains of the GPR1 residues Y15, L19, and Y21 form a hydrophobic core with the chemerin residue F37 (Fig. 1K). The requirement of the GPR1 N-terminus for the chemerin-induced receptor activation was verified by measuring the recruitment of β-arrestin 1 (βarr1) using a bioluminescence resonance energy transfer assay, given that the G protein signaling of GPR1 is weak (Fig. 1B). The data show that removing over 19 residues in the receptor N-terminus reduces the potency of chemerin by over fourfold (Fig. 1L; Table S1). In addition to the interactions formed by the receptor N-terminus, ECL2 also stabilizes the binding between the receptor and chemerin core by not only interacting with the helical region prior to the C-terminus in chemerin but also making close contact with the receptor N-terminus (Fig. 1K). Furthermore, its interactions with both the core and C-terminal regions of chemerin play a role in defining the binding pose of the ligand (Fig. 1K).

Although the cryo-EM map of the chemerin–CMKLR1–G_i1_ complex does not allow modeling of the receptor N-terminus, our functional data imply the importance of this region in mediating the chemerin-induced CMKLR1 activation as well. It was observed that truncating over 20 residues in the receptor N-terminus led to an over 260-fold reduction of chemerin potency in triggering G_i_ activation (Fig. 1O; Table S1). This aligns with previous mutagenesis data suggesting that the negatively charged residues D16, E17, and D20 in the N-terminus of CMKLR1 interact with a positively charged patch of chemerin formed by R94, K95, R96, and K97 (Kretschmer et al., 2023). Despite the involvement of the receptor N-terminus in chemerin binding for both CMKLR1 and GPR1, the poor sequence similarity of the N termini in these two receptors suggests distinct receptor-chemerin interaction patterns in this region (Fig. S4).

Although chemerin exhibits distinct binding poses upon coupling to CMKLR1 and GPR1, its C-terminus activates these two receptors with similar mechanisms (Fig. 1J). In the G_i1_-bound CMKLR1 and GPR1 structures, the residue F156 of chemerin resides at the bottom of the ligand-binding pocket and forms hydrophobic contacts with the tyrosine residue at position 6.51 in both of the receptors (CMKLR1, Y276^6.51^; GPR1, Y262^6.51^; superscripts indicate Ballesteros-Weinstein nomenclature; Fig. 1J and 1M). Compared to the LRH7-C2-bound CMKLR1 structure, this interaction in the chemerin–CMKLR1–G_i1_ and chemerin–GPR1–G_i1_ complexes induces a rearrangement of the hydrophobic core formed by Y276^6.51^, H277^6.52^, and W273^6.48^ in the receptor, resulting in a shift of the “toggle switch” W273^6.48^ and triggering an outward movement of the receptor helix VI on the intracellular side (Figs. 1M, 1N, S5A and S5B). The cryo-EM map of the CMKLR1–LRH7-C2 complex reveals that the histidine residue at the N-terminus of LRH7-C2 obstructs the movement of Y256^6.51^ towards H277^6.52^. Consequently, the three aromatic residues-Y276^6.51^, H277^6.52^, and W273^6.48^-pack more compactly, stabilizing W273^6.48^ in the inactive conformation (Fig. 1N). In addition to the rearrangement of the conserved “toggle switch” residue W273^6.48^ within the CW^6.48^xP motif, the residue F^6.44^ in P^5.50^I^3.40^F6^.44^ motif displays rotamer conformational change upon CMKLR1 activation, triggering the signaling cascade to induce the shift of helix VI and VII (Fig. S5).

In the AlphaFold2-predicted inactive GPR1 model, the non-conserved residue H135^3.50^ (R137^3.50^ in CMKLR1) forms a cation-π or π-π interaction with the residue H244^6.33^ (P258^6.33^ in CMKLR1) to stabilize the inward positioning of helix VI (Fig. S6). Thus, the inactive conformation of GPR1 is more stable and the receptor is unfavorable for G protein binding, as evidenced by our functional data and the relatively poor densities of the chemerin core and the Gα C-terminus in the cryo-EM map of the chemerin–GPR1–G_i1_ complex (Figs. 1R and S6). We further performed mutagenesis studies to assess the importance of these residues in modulating G protein activation. The CMKLR1 mutant containing two mutations R137^3.50^H and P258^6.33^H displays a 60% reduction of the maximal response and a 13-fold drop of agonist potency compared to the wild-type receptor in the chemerin-induced G_i1_ activation assay (Fig. 1S; Table S1). These data suggest differences in residue side chains in some key regions may account for the distinct signaling profiles of CMKLR1 and GPR1.

The outward movement of the receptor helix VI in the G_i1_-bound CMKLR1 and GPR1 generates a cavity on the receptor intracellular surface for G protein binding (Fig. 1I and 1P). Despite the similar active conformations of CMKLR1 and GPR1, the G_i1_ protein adopts distinct binding poses in these two receptors (Fig. 1P–R). The chemerin–CMKLR1–G_i1_ structure adopts a conserved G protein binding conformation similar to those of most class A GPCRs (Fig. 1Q and 1R). While in the GPR1 complex, the G_i1_ protein exhibits an atypical binding mode to the receptor, with the α5 helix moving towards ICL1, resulting in a 10-degree rotation of the αN helix relative to that in the chemerin–CMKLR1–G_i1_ structure (Fig. 1P and 1Q). The distinct binding poses of the α5 helix are likely due to the substitutions of the CMKLR1 residues S140^3.53^ and V141^3.54^ with two larger residues H138^3.53^ and L139^3.54^ in GPR1, which form a spatial hindrance and push the α5 helix away (Fig. 1Q). This movement of the α5 helix leads to a shift of the rest of the G_i1_ protein away from the receptor in the chemerin–GPR1–G_i1_ complex, excluding the interactions between ICL1 and helix VIII of the receptor and the Gβ subunit that are observed in the G_i1_-bound CMKLR1 structure, and weakening the binding between GPR1 and the G_i1_ protein (Fig. 1P). This is supported by our mutagenesis data that introducing an additional mutation S140^3.53^H to the GPR1 mutant R137^3.50^H/P258^6.33^H abolishes the G_i1_ activation (Fig. 1S; Table S1). These structural and functional data suggest that the distinct G protein binding modes may be associated with the differential G protein signaling of CMKLR and GPR1.

Collectively, our research provides a comprehensive elucidation of the molecular determinants for the ligand recognition, activation, and signaling of the chemerin receptors CMKLR1 and GPR1. These findings offer new opportunities for developing therapeutic agents for the treatment of chemerin-related diseases.

## Supplementary data

Supplementary data is available at *Protein & Cell* Journal online https://doi.org/10.1093/procel/pwae073.

pwae073_suppl_Supplementary_Tables_S1-S2_Figures_S3-S6

## Footnotes

The cryo-EM studies were performed at the electron microscopy facility of the Shanghai Institute of Materia Medica (SIMM), Chinese Academy of Sciences. We thank Q. Wang from SIMM for cryo-EM data collection.

The authors declare no competing interests. All data and materials generated or analyzed in this study are available from the corresponding authors. No AI tools had been used in this work. X.L. developed protein expression and purification procedures, prepared protein samples of the chemerin–CMKLR1–G_i1_ and LRH7-C2–CMKLR1 complexes for cryo-EM studies, collected cryo-EM data, performed functional assays, and helped with manuscript preparation. L.Z. developed protein expression and purification procedures, prepared protein samples of the chemerin–GPR1–G_i1_ complex for cryo-EM studies, collected cryo-EM data, and prepared the protein sample of chemerin for functional assays. H.C. performed cryo-EM data processing and analysis, model building and structure refinement. Y.T., X.C., T.L. and M.W. helped with protein preparation and functional assays. C.Y., L.M., and X.C. expressed the proteins. S.H. helped with structure determination and data analysis. Q.Z., B.W., M.H. and Y.Z. initiated the project, planned and analyzed experiments, supervised the research, and wrote the manuscript with input from all coauthors.

This work was supported by the National Key R&D Program of China 2022YFA1302900 (B.W. and S.H.), National Natural Science Foundation of China grants 82121005 (B.W.) and 32401012 (M.H.), CAS Strategic Priority Research Program XDB37030100 (B.W. and Q.Z.), Shanghai Pilot Program for Basic Research—Chinese Academy of Sciences, Shanghai Branch JCYJ-SHFY-2021-008 (B.W.), Shanghai Science and Technology Development Foundation grant 22QA1412000 (Y.Z.), Zhejiang Provincial Natural Science Foundation of China LQ24C050004 (M.H.), and Research Funds from Hangzhou Institute for Advanced Study, University of Chinese Academy of Sciences 2024HIAS-Y011 (M.H.).
