# Supplementary material for: Structural insights into the distinct ligand recognition and signaling of the chemerin receptors CMKLR1 and GPR1
Source: Protein Cell. 2025 Jan 3;16(5):381–5. doi: 10.1093/procel/pwae073 (PMC12120241; doi:10.1093/procel/pwae073)
Supplement: pwae073_suppl_Supplementary_Tables_S1-S2_Figures_S3-S6 [file pwae073_suppl_supplementary_tables_s1-s2_figures_s3-s6.zip › PAC-24549-ZY-Supplementary Materials.pdf]

<u>Supplemental materials for</u>

Structural insights into the distinct ligand recognition and signaling of the chemerin receptors CMKLR1 and GPR1

Xiaowen Lin[1,2,3#], Lechen Zhao[1,3#], Heng Cai[1,4,5#], Xiaohua Chang[1,3], Yuxuan Tang[1,3], Tianyu Luo[1,3], Mengdan Wu[1,3], Cuiying Yi[2], Limin Ma[2], Xiaojing Chu[2], Shuo Han[2,3], Qiang Zhao[2,3,6]*, Beili Wu[1,2,3,5]*, Maozhou He[1,3]*, Ya Zhu[4]*

[1]School of Pharmaceutical Science and Technology, Hangzhou Institute for Advanced Study, University of Chinese Academy of Sciences, Hangzhou, China.

[2]State Key Laboratory of Drug Research, State Key Laboratory of Chemical Biology, Shanghai Institute of Materia Medica, Chinese Academy of Sciences, Shanghai, China.

[3]University of Chinese Academy of Sciences, Beijing, China.

[4]Lingang Laboratory, Shanghai, China.

[5]School of Life Science and Technology, ShanghaiTech University, Shanghai, China.

[6]Zhongshan Institute for Drug Discovery, Shanghai Institute of Materia Medica, Chinese Academy of Sciences, Zhongshan, China.

[7]These authors contributed equally: Xiaowen Lin, Lechen Zhao, Heng Cai

*e-mail: zhaoq@simm.ac.cn; beiliwu@simm.ac.cn; hemaozhou@ucas.ac.cn; zhuya@lglab.ac.cn

The supplemental materials include:

- **Materials and methods of this paper**
- **Figure S1-S6**
- **Table S1-S2**
- **References for the supplemental materials**

Materials and methods of this paper

(1) Cell lines

Spodoptera frugiperda (Sf9, expression systems) and Trichoplusia ni (High Five, Thermo Fisher Scientific) cells were grown in ESF 921 medium at 27 °C and 120 rpm. HEK293F cells were cultured in Freestyle medium (Gibco) with 5% CO_2 at 37 °C and 120 rpm.

(2) Construct design and protein expression

The genes of the human GPR1, CMKLR1 and chemerin were codon-optimized and synthesized by BGI Genomics for insect cell expression. The wild-type receptor was cloned into a modified pFastBac1 vector (Invitrogen) with the HA signal peptide at the N terminus and a PreScission protease site followed by a 2 × strep tag and a Flag tag at the C terminus. To stabilize the samples for cryo-EM analysis, we made several constructions for expression. For the LRH7-C2–CMKLR1, the C-terminal residues of S339-L373 were truncated. The cytochrome b562RIL (BRIL) protein was inserted into the ICL3 region replacing the residues R251-K254 of CMKLR1. The linker sequences ARRQL and ERARSTL from A2A adenosine receptor were introduced to the N-terminus and C-terminus of BRIL, respectively. Further, mutation F259D was executed to stabilize the sample. For the chemerin-GPR1-G_{i1} complex, the receptor was modified by truncating the C-terminal residues L323–Q355 and cloned into the pFastBac1 vector. To improve protein yield and stability of the G_i-bound complexes, a dominant negative $G\alpha_{i1}$ subunit (DN$G\alpha_{i1}$) was generated by introducing five mutations, S47C, G202T, G203A, A326S and E245A. And the human $G\beta_1$ with an N-terminal 6 × His tag and $G\gamma_2$ were cloned into the pFastBac Dual vector (Invitrogen). Proteins were expressed in HighFive cells at a density of 1.5×10^6 cells per ml at 27 °C using the Bac-to-Bac Baculovirus Expression System (Invitrogen). For G_{i1} bound CMKLR1 or GPR1

complexes, the CMKLR1 (or GPR1), chemerin, DNGα_{i1} and G$\beta_1\gamma_2$ (MOI ratio of 5:2:2:2) were co-expressed in the cells. After 48 h of culture, the cells were harvested by centrifugation and stored at −80 °C until use.

(3) Purification of the G_{i1}-bound CMKLR and GPR1 complexes

The frozen cells from 500 ml of cell culture expressing the chemerin–CMKLR1–G_{i1} or chemerin–GPR1–G_{i1} complex were thawed and suspended in 50 ml of suspension buffer containing 25 mM HEPES, pH 7.5, 150 mM NaCl, 10 mM $MgCl_2$ and EDTA-free protease-inhibitor cocktail tablets (Roche). After 1 h of incubation with 25 mU ml^{-1} apyrase (New England BioLabs), the cell membranes were collected by centrifugation at 30,000*g* for 30 min, and then suspended and solubilized in 50 ml of solubilization buffer containing 25 mM HEPES, pH 7.5, 150 mM NaCl, 10 mM $MgCl_2$, 25 mU ml^{-1} apyrase, 0.5% (w/v) LMNG and 0.1% (w/v) CHS (Sigma) at 4 °C for 3 h. The supernatant was collected by centrifugation at 30,000*g* for 30 min and incubated with 1 ml Strep-Tactin Sepharose resin at 4 °C overnight.

To purify the complexes, the strep resin with immobilized complex proteins was washed with 15 column volumes of washing buffer 1 containing 25 mM HEPES, pH 7.5, 150 mM NaCl, 10 mM $MgCl_2$, 0.01% (w/v) LMNG and 0.001% (w/v) CHS. Then the resin was incubated with 25 mM HEPES, pH 7.5, 150 mM NaCl, 10 mM $MgCl_2$ and 0.25% (w/v) GDN at 4 °C for 2 h to exchange the detergent. After that, the resin was washed with 15 column volumes of washing buffer 2 containing 25 mM HEPES, pH 7.5, 150 mM NaCl, 10 mM $MgCl_2$ and 0.01% (w/v) GDN. The complex proteins were eluted with 200 mM Tris-HCl, pH 8.0, 150 mM NaCl, 10 mM $MgCl_2$, 50 mM biotin and 0.01% (w/v) GDN. The elution was then incubated with 500 µl Ni-NTA resin (Clontech) at 4 °C for 1 h and washed with 10 column volumes of washing buffer 3 containing 25

mM HEPES, pH 7.5, 150 mM NaCl, 10 mM $MgCl_2$, 0.01% (w/v) GDN and 10 mM imidazole. The complex proteins, which were eluted with 25 mM HEPES, pH 7.5, 150 mM NaCl, 10 mM $MgCl_2$, 0.01% (w/v) GDN and 300 mM imidazole, were loaded to size-exclusion chromatography using a Superdex 200 Increase 10/300 column (GE Healthcare) pre-equilibrated with a buffer containing 25 mM HEPES, pH 7.5, 150 mM NaCl, 10 mM $MgCl_2$ and 0.01% (w/v) GDN. The complex fractions were pooled and concentrated to 2.5–3.5 mg ml^{-1} (chemerin–CMKLR1–G_{i1}, 3.5 mg ml^{-1}; chemerin–GPR1–G_{i1}, 2.5 mg ml^{-1}) with a 100-kDa molecular-weight cutoff concentrator (Millipore) for cryo-EM experiments. Protein purity and homogeneity were analyzed by SDS-PAGE, native PAGE, and analytical size-exclusion chromatography using a 4.6 × 250 mm Nanofilm SEC-250 column (Sepax Technologies).

(4) Purification of the LRH7-C2–CMKLR1 complex

Different from the purification procedures of the G_{i1}-bound complexes, the cell pellets expressed CMKLR1 were firstly washed with a hypotonic buffer (10 mM HEPES, pH 7.4, 20 mM KCl, 10 mM MgCl2, EDTA-free protease inhibitor cocktail tablets), and then centrifuged at 14,000*g* for 30 min. The precipitates were kept and washed with a high osmotic buffer (10 mM HEPES, pH 7.4, 1 M NaCl. 20 mM KCl, 10 mM MgCl2, EDTA-free protease inhibitor cocktail tablets). The purified membrane was solubilized in the buffer containing 20 mM HEPES, pH 7.4, 150 mM NaCl, 0.5% (w/v) DDM, 0.1% (w/v) CHS, 100 μM LRH7-C2 and 2mg mL^{-1} iodoacetamide for 2 h at 4 °C. The supernatant was then collected upon ultracentrifugation for 30 mins, and incubated with Strep-Tactin Sepharose resin at 4 °C overnight. Upon washing the resin, CMKLR1 was eluted with 200 mM Tris-HCl, pH 8.0, 150 mM NaCl, 10 mM $MgCl_2$, 50 mM biotin and 0.01% (w/v) GDN. The eluted CMKLR1 was incubated with previously purified anti-BRIL Fab for 2 h at 4 °C. After the second affinity purification using Ni-NTA resin, we got the purified CMKLR1 complexed with

anti-BRIL Fab, which was loaded into the pre-equilibrated Superdex 200 Increase 10/300 column. The fractions from peak were collected and concentrated to ~3 mg mL^{-1} for cryo-EM analysis.

(5) Cryo-EM data acquisition and processing

For cryo-EM analysis, 3 μl of the purified samples were applied onto freshly glow-discharged amorphous NiTi foil R1.2/1.3 300 mesh Au grids. After blotting in Vitrobot Mark IV (Thermo Fisher Scientific) that was maintained at 4 °C with 100% humidity, the grids were plunged into the frozen liquid ethane. The grids were then transferred and stored in liquid nitrogen for further cryo-EM inspection. Data were collected in 300 kV Titan Krios G3 TEM (FEI) equipped with K3 direct electron detector (DED, Gatan Inc.) and a GIF-Quantum energy filter. The nominal magnification was set to ×81,000, resulted in a calibrated physical pixel size of 1.071 Å at specimen level, or 0.5355 Å under super-resolution mode. The slit width was set to 20 eV, and nominal defocus was set to between -0.5 to -1.5 μm. The total exposure time was set to 3 s with a movie of 40 frames. The total exposure dose is 70 electrons per $Å^2$. All images were collected using SerialEM software (Mastronarde, 2003).

For the chemerin–CMKLR1–G_{i1} complex, a total of 9,793 movies were collected. After motion correction and CTF estimation using MotionCor2 (Zheng et al., 2017) and CTFFIND4 (Rohou and Grigorieff, 2015), we picked 11,178,958 particles and extracted with a box size of 256 pixels (1.071 Å per pixel) using template picker in cryoSPARC (Punjani et al., 2017). These particles were subjected to two rounds of 2D classification and one round of heterogeneous refinement. The best classes with 1,134,219 particles were selected for further processing. Then, one more round of heterogeneous refinement were carried out to generate four different classes. The best class with 760,566 particles (~67%) show clear features of chemerin and CMKLR1. To

improve map quality, two different strategies were applied onto the chemerin–CMKLR1 and G_{i1} protein regions. For the chemerin–CMKLR1 part, one round of 3D classification without alignment was performed, 89,786 particles (12%) from the best class with the best features of chemerin and CMKLR1 were further processed using non-uniform refinement, and yielded map with a global resolution at 3.4 Å. To further improve the map quality, a soft mask containing the receptor and chemerin was generated and subjected to local refinement, yielding a map at 3.5 Å resolution. For the G_{i1} protein region, firstly, the aforementioned 760,566 particles dataset were converted to RELION-4 (Kimanius et al., 2021) for Bayesian polishing. The polished particles were then transferred back to CryoSPARC for non-uniform refinement, and a map at 2.8 Å resolution was obtained. Then, local refinement was performed to improve the resolution of the G_{i1} protein region to 2.7 Å. A composited map of the whole complex was generated with ChimeraX (Pettersen et al., 2021).

For the chemerin–GPR1–G_{i1} complex, 10,486 movies were collected and executed following the same processing procedure as the chemerin–CMKLR1–G_{i1} complex. 8,303,102 particles with a box size of 256 pixels (1.071 Å per pixel) were extracted and applied to three rounds of 2D classification and one round of heterogeneous refinement. 1,988,899 particles were then subjected to ab initio refinement and one round of heterogeneous refinement. The best class containing 783,161 particles (39%) were selected. For the chemerin–GPR1 region, these particles were transferred to RELION-4 for 3D classification (K=8 and T=100) without alignment using the local mask. 59,593 particles (8%) showing the best densities of chemerin were selected and subjected to non-uniform refinement and local refinement, yielding a map of the chemerin–GPR1 region at 3.6 Å resolution. For the G_{i1} protein region, the 783,161 particles were polished using RELION-4. After non-uniform refinement, we got a global density map at 3.0 Å resolution. To

further improve the map quality, local refinement was applied using a mask encompassing the G_{i1} protein, resulting in a map at 2.8 Å resolution. A composited map of the complex was generated with ChimeraX.

For the LRH7-C2–CMKLR1, 16,739 movies were collected. Upon motion correction and CTF estimation, 15,044,669 particles were extracted with a box size of 256 pixels (1.071 Å per pixel) using template picker in CryoSPARC. These particles were applied to four rounds of 2D classification and one round of heterogeneous refinement. 570,260 good particles were selected and subjected to ab initio refinement and one round of heterogeneous refinement. The two classes containing 488,139 particles (86%) were selected and refined to a resolution of 3.7 Å using non-uniform refinement. To further improve the density quality of the receptor, the particles were suffered to 3D classification (K=6 and T=4) without alignment, and the best-featured class containing 260,967 particles was selected. Upon polish and 3D refinement in RELION-5 with blush regularization, we significantly improved the local resolution of receptor, and determined the structure at a resolution of 3.9 Å.

All particles were converted from CryoSPARC to RELION or vice versa using pyem (Asarnow, 2019). Resolutions of all the maps were determined using the fourier shell correlation (FSC) cut-off criteria at 0.143 (Scheres and Chen, 2012). Local resolution was determined using ResMap (Kucukelbir et al., 2014).

(6) Model building and refinement

The models of LRH7-C2–CMKLR1, chemerin–CMKLR1–G_{i1} and chemerin–GPR1–G_{i1} complexes were built using the AlphaFold2 (Jumper et al., 2021) predicted models of chemerin, CMKLR and GPR1, and the G_i heterotrimer from the μ-opioid receptor (μOR)–G_i complex

structure (PDB ID: 6DDE) as initial models. All the models were docked into the cryo-EM electron density maps using Chimera (Pettersen et al., 2004), followed by iterative manual adjustments in COOT (Emsley and Cowtan, 2004) and real-space refinement using phenix.real_space_refine in Phenix (Adams et al., 2010). The models were validated using Molprobity (Chen et al., 2010). Structural figures were prepared by Chimera or PyMOL (DeLano, 2002) (https://pymol.org/2/). The data collection and structure refinement statistics are provided in Table S2.

(7) Purification of chemerin

Human chemerin (residues E21–S157) was cloned into the pET32a vector (Sango Biotech) with an N-terminal ThioredoxinA (TrxA) and 10 × His tags followed by a S tag and a PreScission protease site. The plasmid was transformed into the BL21/DE3 competent *Escherichia coli* cells and cultured at 37 °C to OD600 of 0.6–0.8. Then the cells were induced with 1 mM IPTG at 37 °C for 4 h, and harvested by centrifugation. The cell pellets were resuspended in the lysis buffer (25 mM Tris, pH 8.0, 300 mM NaCl and protease inhibitor). The resuspended cells were lysed by sonication and centrifuged at 14,000*g* for 30 min at 4 °C. The precipitates were kept and was washed with the wash buffer (25 mM Tris, pH 8.0, 300 mM NaCl and 2 M Urea). The washed pellets were solubilized with the denaturing buffer (25 mM Tris, pH 8.0, 300 mM NaCl and 8 M urea). The supernatants were collected and incubated with Ni-NTA resin for 1 h at 4 °C. The proteins were then eluted with 25 mM Tris, pH 8.0, 300 mM NaCl, 6 M urea and 300 mM imidazole upon washing with the buffer containing 25 mM Tris, pH 8.0, 300 mM NaCl, 6 M urea and 30 mM imidazole. The eluted chemerin was refolded in a series of refolding buffers with decreased concentrations of urea. Firstly, the eluted chemerin was dialyzed against the refolded buffer (25 mM Tris, pH 8.0, 300 mM NaCl, 0.44 mM KCl, 2 mM $MgCl_2$, 2 mM $CaCl_2$, 500 mM L-arginine, 0.055% polyethylene glycol 8000, 5% glycerol, 1 mM reduced L-glutathione and 0.1

mM oxidized L-glutathione) supplemented with 4 M urea for 12 h at 4 °C, then transferred to the refolded buffer supplemented with 2 M urea for 12 h at 4 °C. The samples were finally dialyzed into the buffer without urea (25 mM Tris, pH 8.0, 300 mM NaCl) for 12 h at 4 °C, and cleaved off the N-terminal tags with HRV 3C PreScission Protease overnight at 4 °C. The final chemerin were purified with Ni-NTA resin and concentrated to 0.6−6 mg ml^{-1}. The samples were aliquoted and stored at −80 °C.

(8) BRET assay using TRUPATH biosensors

To measure the chemerin or C9 peptide induced G_i activation of CMKLR1, a BRET assay was performed using TRUPATH biosensors obtained from Addgene (TRUPATH Kit, #1000000163). For the arrestin binding assay, the Rluc8 donor was added to the C termini of the wild-type GPR1 and mutants, and the GFP2 acceptor were fused to the human βarr1 and βarr2. The Flag-tagged receptors and the arrestins were cloned into the pTT5 vector (Invitrogen). Two milliliters of HEK293F cells (Invitrogen) were transiently co-transfected with a total of 2 μg plasmids for CMKLR1 (or mutants), $G\alpha_{i1}$-RLu8, $G\beta_3$ and $G\gamma_9$-GFP2 at an MOI ratio of 1:1:1:1 and 4 μg plasmids of GPR1-Rluc8 (or mutants) and βarr1-GFP2 (or βarr2-GFP2) at an MOI ratio of 1:1. The cells were harvested after 48 h post transfection and the cell-surface expression of the receptors was measured by mixing 10 μl cells with 15 μl of TBS buffer supplemented with 4% BSA, 20% (v/v) viability-staining solution 7-AAD (Invitrogen, Cat#00-6993-50) and 0.1% (v/v) anti-FLAG M2-FITC antibody (Sigma, Cat#F4049) at 4 °C for 20 min. Then the mixture was diluted by adding 175 μl of TBS buffer, and the fluorescent signal was measured using a flow-cytometry reader (Guava easyCyte HT, Millipore).

The cells were plated into the 96-well opaque cell culture plates (Beyotime) at a density of 30,000 cells per well in 60 μl of BRET2 assay buffer containing 1× Hanks' balanced salt solution (HBSS, Gibco) and 20 mM HEPES, pH 7.4. After incubating for 30 min at 37 ℃, 10 μl of freshly prepared 50 μM coelenterazine 400a (Nanolight Technologies) was added into each well. After 5–10 min, the BRET baselines were read by the Synergy II (Bio-Tek) plate reader with 410 nm (RLuc8-coelenterazine 400a) and 515 nm (GFP2) emission filters for 16 min. Then 30 μl chemerin at different concentrations (1 pM–10 μM) were added into the wells and the BRET signals were monitored continuously five times. The BRET ratios were calculated as the ratio of the GFP2 emission to RLuc8 emission and the data were analyzed using GraphPad Prism 8.0.

(9) Data availability

Atomic coordinates and cryo-EM density maps for the structures of chemerin–CMKLR1–G_{i1}, chemerin–GPR1–G_{i1} and the LRH7-C2–CMKLR1 complexes have been deposited in the PDB under identification codes 9L3W, 9L3Y and 9L3Z, respectively, and in the Electron Microscopy Data Bank under accession codes EMD-62730 (consensus map for chemerin–CMKLR1–G_{i1}), EMD-62732 (focused refinement for chemerin–CMKLR1 region of chemerin–CMKLR1–G_{i1}), EMD-62792 (focused refinement for G protein region of chemerin–CMKLR1–G_{i1}), EMD-62793 (composite map for chemerin–CMKLR1–G_{i1}), EMD-62794 (consensus map for chemerin–GPR1–G_{i1}), EMD-62796 (focused refinement for chemerin–GPR1 region of chemerin–GPR1–G_{i1}), EMD-62795 (focused refinement for G protein region of chemerin–GPR1–G_{i1}), EMD-62798 (composite map for chemerin–GPR1–G_{i1}) and EMD-62799 (cryo-EM map for LRH7-C2–CMKLR1), respectively.

Supplementary Figures

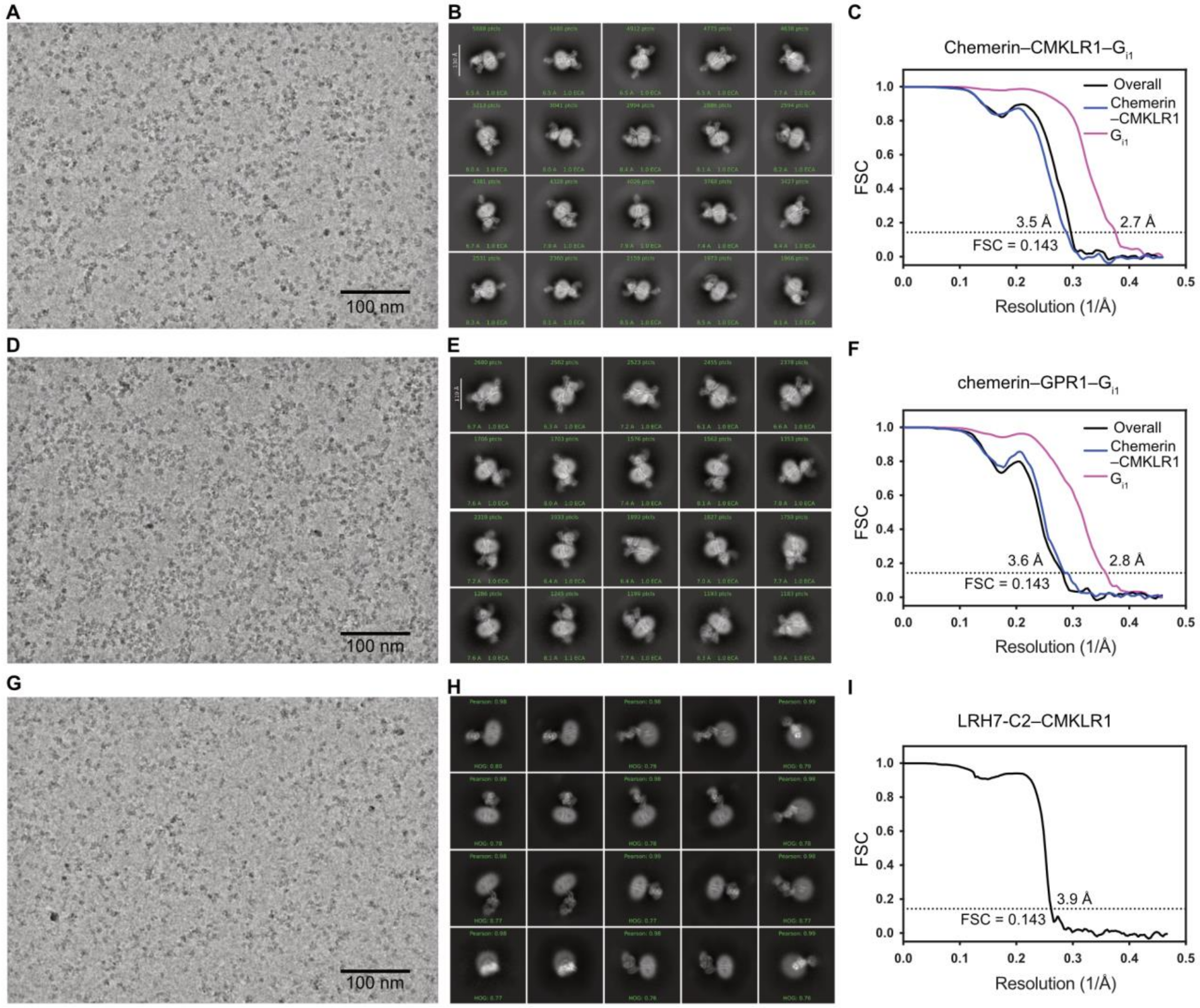

Figure S1. Cryo-EM analysis of the GPR1 and CMKLR1 complexes.

(**A**, **D and G**) Representative cryo-EM images of the chemerin−CMKLR1−G_{i1}, chemerin−GPR1−G_{i1} and LRH7-C2–CMKLR1 complexes. **(B, E and H**) 2D averages of the complexes. (**C, F and I)** Gold-standard Fourier shell correlation (FSC) curves of the complexes.

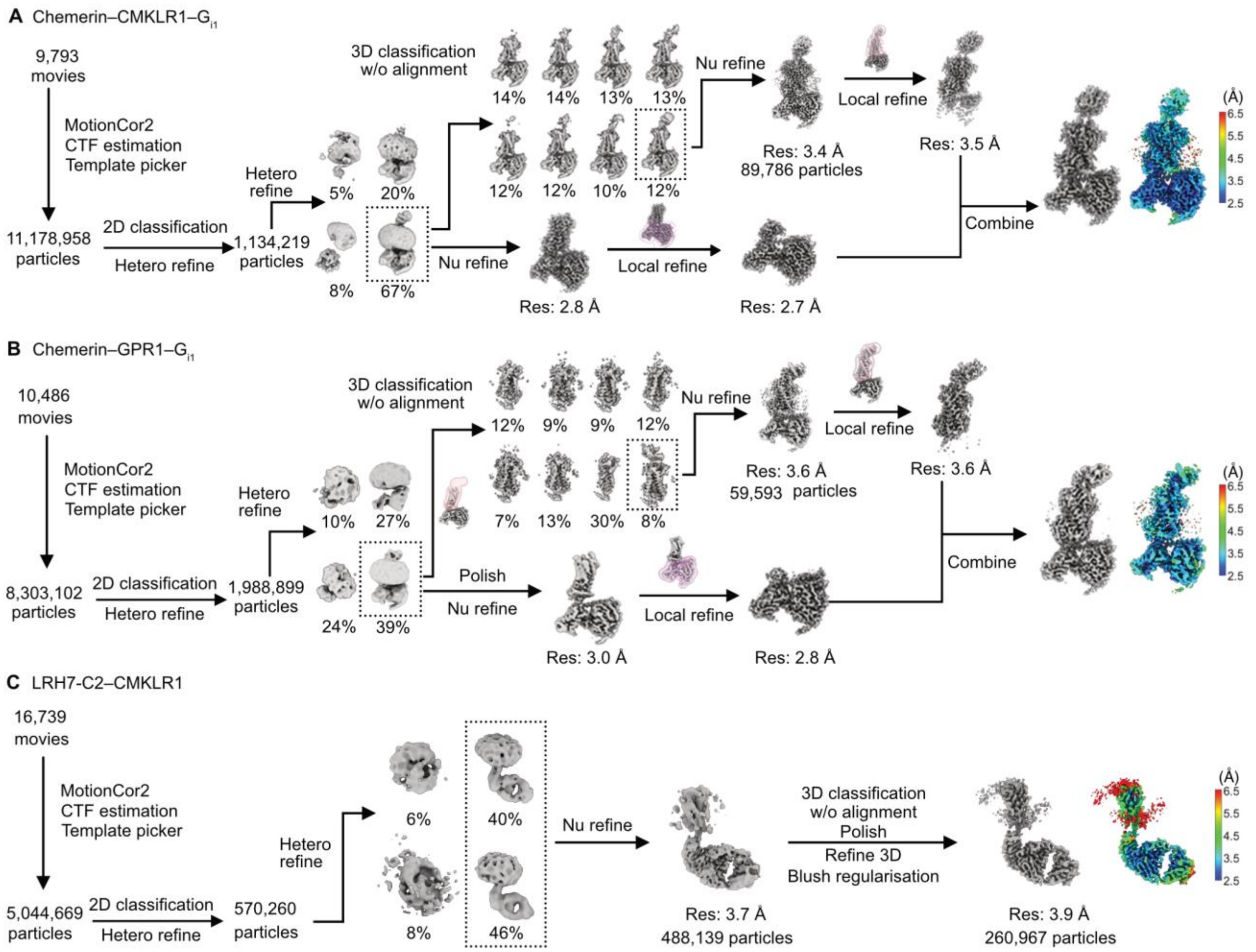

Figure S2. Workflow of cryo-EM data processing.

(**A**) Cryo-EM data processing workflow of CMKLR1–chemerin–G_{i1}. **(B)** Cryo-EM data processing workflow of GPR1–chemerin–G_{i1}. **(C)**. Cryo-EM data processing workflow of LRH7-C2–CMKLR1. The final cryo-EM maps are colored according to local resolution (in Å). To simplify the workflow, the terms ‘heterogeneous refinement’, ‘non-uniform refinement’, and ’Bayesian polishing’ are abbreviated as ‘Hetero refine’, ‘Nu refine’, and ‘Polish’, respectively.

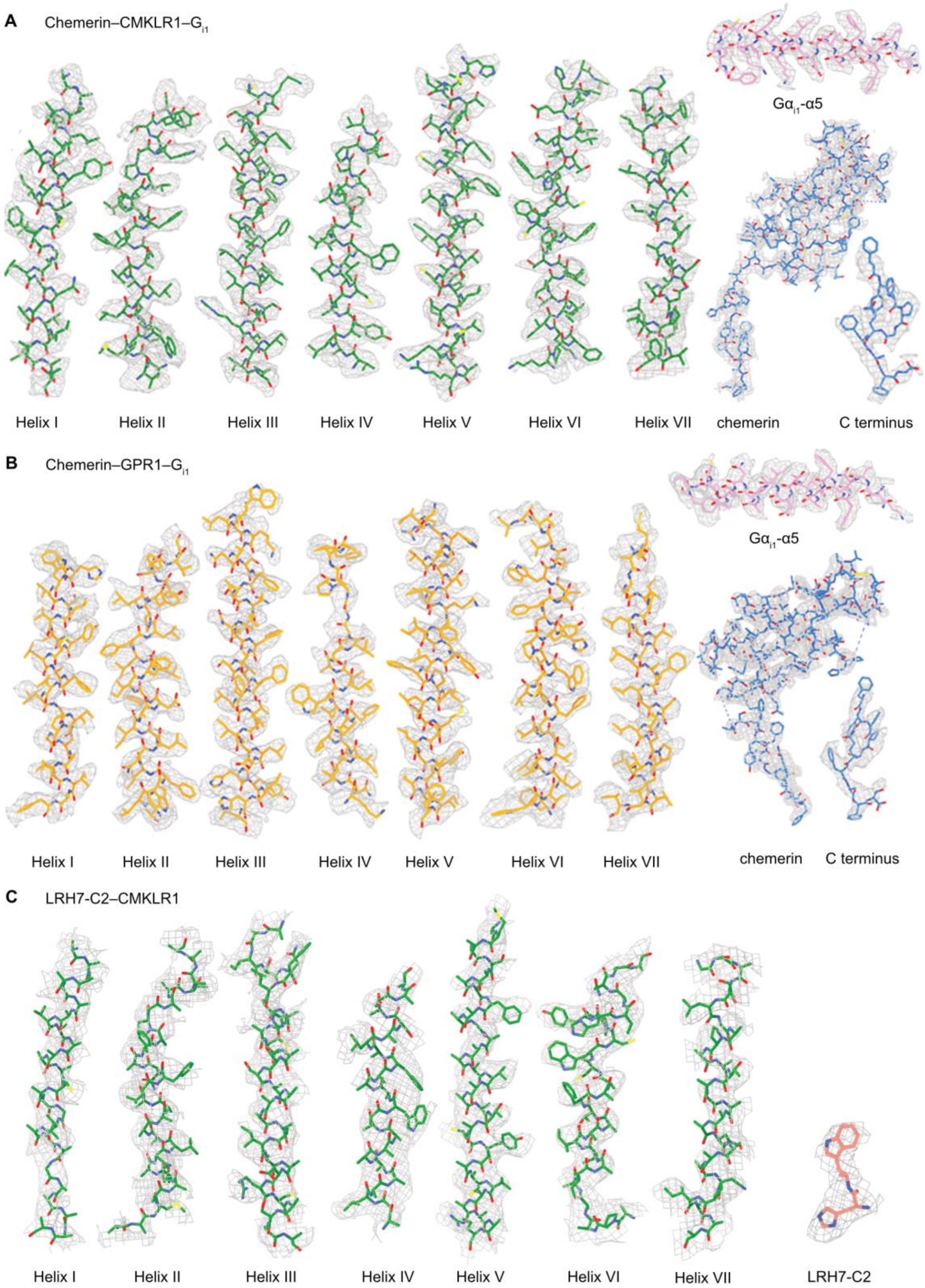

Figure S3. Cryo-EM maps of the CMKLR1 and GPR1 structures.

(**A**) Cryo-EM density map and model of chemerin–GPR1–G_{i1}. **(B)** Cryo-EM density map and model of chemerin–CMKLR1–G_{i1} complexes. **(C)** Cryo-EM density map and model of LRH7-C2–CMKLR1. The Cryo-EM density maps and models of chemerin–GPR1–G_{i1} and chemerin–CMKLR1–G_{i1} are displayed for all receptor transmembrane helices, the α5-helix in $G\alpha_{i1}$ ($G\alpha_{i1}$-α5) and chemerin. The maps are colored grey. The models are shown as sticks and colored according to chains.

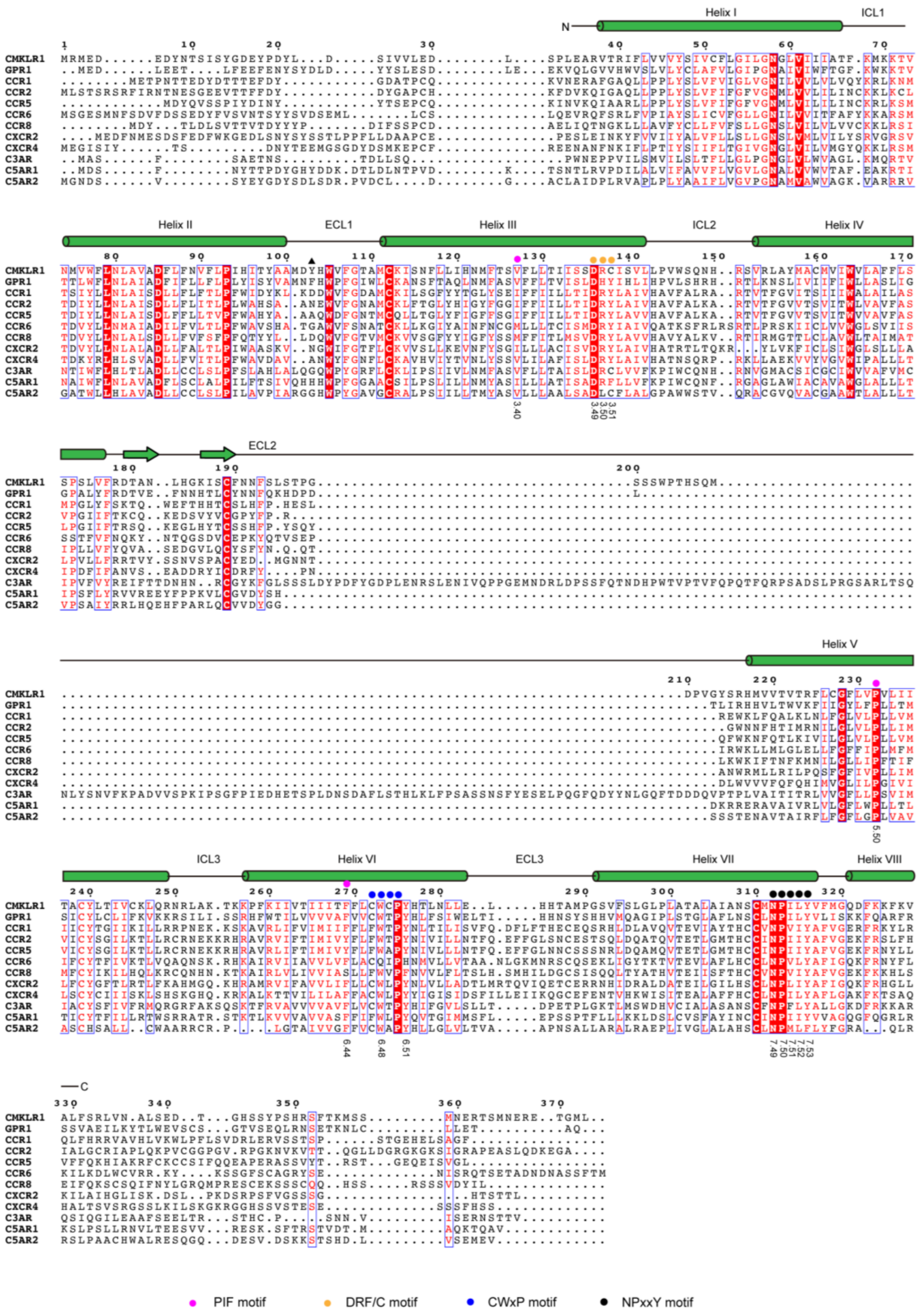

Helix I
ICL1
Helix II
ECL1
Helix III
ICL2
Helix IV
ECL2
Helix V
ICL3
Helix VI
ECL3
Helix VII
Helix VIII
CMKLR1
GPR1
CCR1
CCR2
CCR5
CCR6
CCR8
CXCR2
CXCR4
C3AR
C5AR1
C5AR2
PIF motif
DRF/C motif
CWxP motif
NPxxY motif

Figure S4. Multiple sequence alignment of CMKLR1, GPR1 and other class A GPCRs.

Sequence alignments were generated using Clustal W program. The diagram was prepared on the ESPript 3.0 server (http://espript.ibcp.fr/ESPript/cgi-bin/ESPript.cgi). The secondary-structures were depicted according to the chemerin–CMKLR1–G_{i1} structure.

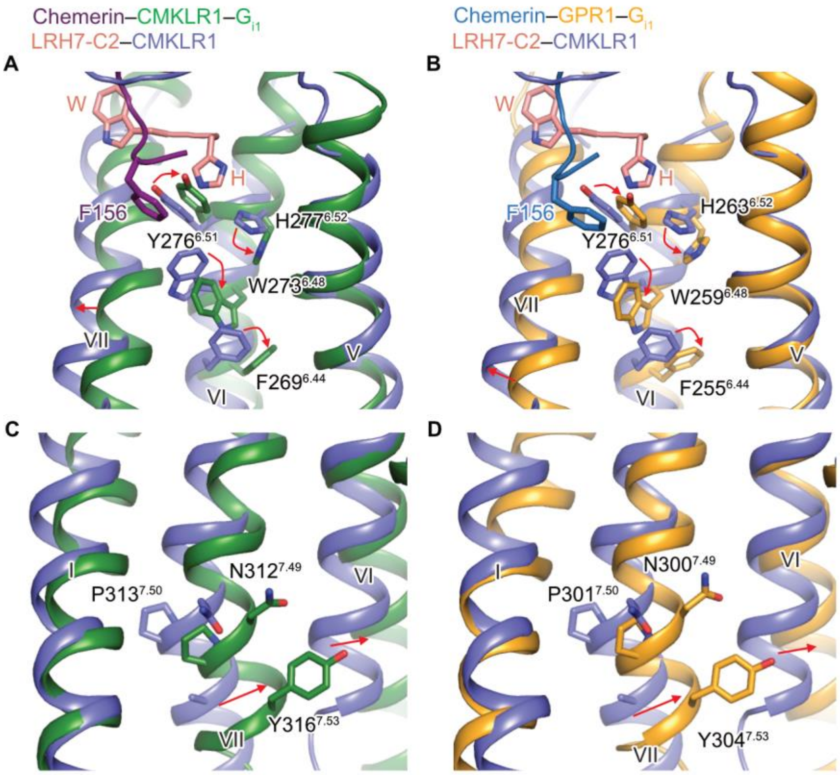

Figure S5. Chemerin induced CMKLR1 and GPR1 signal transduction.

(A and **B)** Comparison of conformational change of toggle switch between inactive CMKLR1 and CMKLR1 or GPR1. **(C** and **D)** Comparison of conformational change of helix VI and VII between inactive CMKLR1 and CMKLR1 or GPR1**.** In the structure of chemerin–CMKLR1–G_{i1}, chemerin is colored by deep purple, CMKLR1 by forest green, and shown as cartoon representation. In the structure of chemerin–GPR1–$G_{i1,}$ chemerin is colored by sky blue, GPR1 by orange, and shown as cartoon representation. In the inactive state of CMKLR1 structure, antagonist LRH7-C2 is colored by salmon, CMKLR1 by slate blue, and shown as cartoon representation. The residues involved in these motifs are shown as sticks. The translation and rotation are indicated as red arrows.

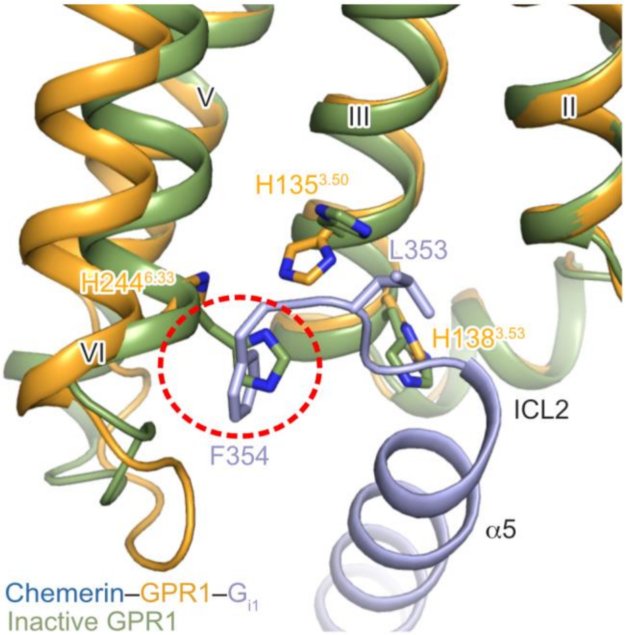

Figure S6. Comparison of the active and predicted inactive states of GPR1 structures.

Superposition of the predicted inactive GPR1 and chemerin–GPR1–G_{i1} structures, showing the steric clash between inactive GPR1 and G_{i1} α5 helix. The clashed region is highlighted by a red dashed circle. The active and inactive states of GPR1 are colored as bright orange and green, respectively. The G protein was colored as light blue. The proposed key residues that are involved in interactions are shown as sticks**.**

References

Adams, P.D., Afonine, P.V., Bunkóczi, G., Chen, V.B., Davis, I.W., Echols, N., Headd, J.J., Hung, L.-W., Kapral, G.J., and Grosse-Kunstleve, R.W. (2010). PHENIX: a comprehensive Python-based system for macromolecular structure solution. Acta Crystallographica Section D: Biological Crystallography *66*, 213-221.

Asarnow, D., Palovcak, E., and Cheng, Y. (2019). UCSF pyem v0. 5. Zenodo. https://doi.org/10.5281/zenodo.3576630.

Chen, V.B., Arendall, W.B., Headd, J.J., Keedy, D.A., Immormino, R.M., Kapral, G.J., Murray, L.W., Richardson, J.S., and Richardson, D.C. (2010). MolProbity: all-atom structure validation for macromolecular crystallography. Acta Crystallographica Section D: Biological Crystallography *66*, 12-21.

DeLano, W.L. (2002). Pymol: An open-source molecular graphics tool. CCP4 Newsl Protein Crystallogr *40*, 82-92.

Emsley, P., and Cowtan, K. (2004). Coot: model-building tools for molecular graphics. Acta crystallographica section D: biological crystallography *60*, 2126-2132.

Jumper, J., Evans, R., Pritzel, A., Green, T., Figurnov, M., Ronneberger, O., Tunyasuvunakool, K., Bates, R., Žídek, A., and Potapenko, A. (2021). Highly accurate protein structure prediction with AlphaFold. nature *596*, 583-589.

Kimanius, D., Dong, L., Sharov, G., Nakane, T., and Scheres, S.H. (2021). New tools for automated cryo-EM single-particle analysis in RELION-4.0. Biochemical Journal *478*, 4169-4185.

Kucukelbir, A., Sigworth, F.J., and Tagare, H.D. (2014). Quantifying the local resolution of cryo-EM density maps. Nature methods *11*, 63-65.

Mastronarde, D.N. (2003). SerialEM: a program for automated tilt series acquisition on Tecnai microscopes using prediction of specimen position. Microscopy and Microanalysis *9*, 1182-1183.

Pettersen, E.F., Goddard, T.D., Huang, C.C., Couch, G.S., Greenblatt, D.M., Meng, E.C., and Ferrin, T.E. (2004). UCSF Chimera—a visualization system for exploratory research and analysis. Journal of computational chemistry *25*, 1605-1612.

Pettersen, E.F., Goddard, T.D., Huang, C.C., Meng, E.C., Couch, G.S., Croll, T.I., Morris, J.H., and Ferrin, T.E. (2021). UCSF ChimeraX: Structure visualization for researchers, educators, and developers. Protein science *30*, 70-82.

Punjani, A., Rubinstein, J.L., Fleet, D.J., and Brubaker, M.A. (2017). cryoSPARC: algorithms for rapid unsupervised cryo-EM structure determination. Nature methods *14*, 290-296.

Rohou, A., and Grigorieff, N. (2015). CTFFIND4: Fast and accurate defocus estimation from electron micrographs. Journal of structural biology *192*, 216-221.

Scheres, S.H., and Chen, S. (2012). Prevention of overfitting in cryo-EM structure determination. Nature methods *9*, 853-854.

Zheng, S.Q., Palovcak, E., Armache, J.-P., Verba, K.A., Cheng, Y., and Agard, D.A. (2017). MotionCor2: anisotropic correction of beam-induced motion for improved cryo-electron microscopy. Nature methods *14*, 331-332.

Table S1. Arrestin binding and G_{i1} activation of GPR1 and CMKLR1 measured by BRET and IP accumulation assays

Chemerin-induced βarr1 binding of GPR1

	WT/mutants[a]	EC_{50} (nM)	pEC_{50}[b]	E_{max}[c]	n[d]	Expression[e] (% of WT)
GPR1	WT	13	7.89 ± 0.06	100 ± 3	26	100
	WT-C9[f]	13	7.90 ± 0.15	46 ± 3***	3	100
	△M1-E11	25	7.60 ± 0.14	96 ± 5	3	63 ± 5
	△M1-L19	95	7.02 ± 0.19*	113 ± 10	3	62 ± 4
	△M1-Y22	218	6.66 ± 0.22***	100 ± 10	3	131 ± 19
	△M1-E25	247	6.61 ± 0.20***	95 ± 9	3	60 ± 5
	△M1-L28	104	6.98 ± 0.33*	101 ± 16	3	55 ± 6
	△M1-E29	nd	nd	nd	3	58 ± 7
CMKLR1	WT	6.48	8.19±0.21	84±7	7	83±6
CMKLR1	WT	6.48	8.19±0.21	100 ± 4	7	100
	WT-C9[f]	513	6.29 ± 0.13***	61 ± 3***	3	100

Chemerin-induced G_i activation of CMKLR1

	WT/mutants[a]	EC_{50} (nM)	pEC_{50}[b]	E_{max}[c]	n[d]	Expression[e] (% of WT)
CMKLR1	WT	3.6	8.45 ± 0.06	100 ± 2	14	100
	WT-C9[f]	304	6.52 ± 0.12***	81 ± 4	3	100
	△M1-T10	27	7.57 ± 0.22*	86 ± 7	3	55 ± 6*
	△M1-D20	942	6.02 ± 0.25***	61 ± 9***	3	54 ± 9*
	△M1-I25	1,258	5.90 ± 0.32***	47 ± 9***	4	49 ± 3**
	$R137^{3.50}H+P258^{6.33}H$	49	7.31 ± 0.19***	40 ± 3***	4	160 ± 6***
	$R137^{3.50}H+S140^{3.53}H+P258^{6.33}H$	nd	nd	nd	3	121 ± 12
GPR1	WT	nd	nd	nd	3	93 ± 32

C9 or chemerin-induced IP accumulation of GPR1

	WT	Span[c,d] (% of WT)	n[d]	Expression[e] (% of WT)
GPR1	WT	100	3	100
	WT-C9	87± 9	3	100
	WT-chemerin	468± 61**	3	100

[a]All mutations were introduced in the wild-type (WT) receptor.

[b]Data are shown as mean ± SEM from at least three independent experiments performed in duplicate. $*P < 0.05$, $**P < 0.001$, $***P < 0.0001$ (one-way ANOVA followed by Dunnett's post-test, compared with the response of WT).

[c]The maximal response is reported as a percentage of the maximum effect of chemerin-induced response at the WT. nd, not determined (data for which a robust concentration response curve could not be established within the concentration range tested, such that an E_{max} was not reached).

[d]Sample size, the number of independent experiments performed in triplicate.

[e]Protein expression levels of CMKLR1 and GPR1 constructs at the cell surface were determined in parallel by flow cytometry with an anti-FLAG antibody and reported as per cent compared to the wild type from at least three independent measurements performed in duplicate.

[f]C9-induced βarr1 recruitment or G_i activation of GPR1 or CMKLR1.

Table S2. Cryo-EM data collection, refinement and validation statistics.

	**Chemerin–CMKLR1–G_{i1} (PDB 9L3W) (EMDB-62793)**	**Chemerin–GPR1–G_{i1} (PDB 9L3Y) (EMDB-62798)**	**LRH7-C2–CMKLR1 (PDB 9L3Z) (EMDB-62799)**
Magnification	81,000	81,000	81,000
Voltage (kV)	300	300	300
Electron exposure ($e^{–}/Å^2$)	70	70	70
Defocus range (μm)	–0.8 ~ –1.5	–0.8 ~ –1.5	–0.8 ~ –1.5
Pixel size (Å)	1.071	1.071	1.071
Symmetry imposed	C1	C1	C1
Initial particle images (no.)	11,178,958	8,303,102	15,044,669
Final particle images (no.)	89,786	59,593	260,967
Map resolution (Å)	3.5 (chemerin–CMKLR1)<br>2.7 (G_{i1})	3.6 (chemerin–GPR1)<br>2.8 (G_{i1})	3.9
**Refinement**<br>FSC threshold	0.143	0.143	0.143
Map resolution range (Å)	2.6-6.5	2.5-6.5	2.5-6.5
Initial model used (PDB code)	6DDE	6DDE	Alphafold2
Model resolution (Å)	3.2	3.4	4.1
FSC threshold	0.5	0.5	0.5
Map sharpening *B* factor ($Å^2$)	–96 (chemerin–CMKLR1)<br>–86 (G_{i1})	–108 (chemerin–GPR1)<br>–89 (G_{i1})	–143
Model composition			
Non-hydrogen atoms	7,262	7,383	5147
Protein residues	983	993	734
Lipid	0	0	0
B factors ($Å^2$)			
Protein	55.44	52.38	30.04
Lipid	/	/	/
R.m.s. deviations			
Bond lengths (Å)	0.002	0.002	0.004
Bond angles (°)	0.515	0.565	0.970
Validation			
MolProbity score	1.55	1.86	1.78
Clashscore	6.66	7.84	6.73
Poor rotamers (%)	0.00	0.00	0.21
Ramachandran plot			
Favored (%)	96.96	93.35	93.84
Allowed (%)	3.04	6.65	6.16
Disallowed (%)	0.00	0.00	0.00
